# Urinary Tract Infection in Chronic Kidney Disease Population: A Clinical Observational Study

**DOI:** 10.7759/cureus.12486

**Published:** 2021-01-04

**Authors:** Mythri Shankar, Shashikala Narasimhappa, Madhura N.S.

**Affiliations:** 1 Nephrology, Institute of Nephro-Urology, Bengaluru, IND; 2 Microbiology, Institute of Nephro-Urology, Bengaluru, IND; 3 Biochemistry, Institute of Nephro-Urology, Bengaluru, IND

**Keywords:** microbiological culture, chronic kidney disease, drug resistance, uropathogens

## Abstract

Introduction

Chronic kidney disease (CKD) is a state of chronic inflammation. Chronic inflammation weakens the body's immune response to infections. Hence, CKD patients are at an increased risk of infections. Urinary tract infection (UTI) is one of the most common types of community-acquired infection. There is a paucity of data with respect to UTI in CKD patients. Hence, our objective was to study the clinical and microbiological profile of UTI in CKD patients.

Materials and methods

We studied 129 CKD patients at a tertiary care hospital in south India from January 2020 to June 2020. Patients who fulfilled the inclusion criteria were included in the study. Urine samples were cultured aseptically. Only urine-culture positive samples were included in the study and antibiotic susceptibility was recorded.

Results

Males (76.2%) were most commonly infected. 94% were gram-negative bacteria, 3% were gram-positive bacteria and 3% were Candida species. E. coli (61.8%) was the most common isolated microorganism. Resistance to quinolones was recorded among gram-negative bacteria. Resistance to penicillin and quinolones was noted among gram-positive bacteria. Candida species were sensitive to amphotericin B and fluconazole.

Conclusion

The results of the study help in formulating the empiric antibiotic policy to treat UTI in CKD patients and hence prevent inadvertent use of antibiotics and the emergence of antibiotic resistance.

## Introduction

Chronic kidney disease (CKD) is a major public health problem to handle in developing nations like India [1]. Patients with CKD usually have other comorbidities, such as diabetes mellitus, hypertension, which adds to both financial burden as well as increase in morbidity [1]. Complications of CKD are reduced immunity, anemia, malnutrition, inflammation, vitamin deficiencies and poor quality of life [2]. Also, patients on prolonged hemodialysis have reduced immunity and are more prone to infections such as urinary tract infection (UTI) [3].

UTI is pathological microbial invasion of the urinary tract. It is one of the most common infections affecting people in the community and hospitals. Approximately 150 million people every year are affected with UTI [4]. Majority of UTIs are of bacterial etiology [4]. UTI has been diagnosed by urine culture since decades [4]. In studies conducted in different parts of India, the prevalence varies from 21.8% to 31.3% [5]. UTI, if left untreated, can progress to worsen renal function, cause pyelonephritis, sepsis, septic shock and death. Hence, it is important to treat UTI in earlier stages to prevent significant morbidity and mortality [6]. 

Data from UTI in animal studies state that an adequate concentration of antibiotics in urine is required to treat the infection [3]. This depends on the serum concentration of the drug, the glomerular filtration rate (GFR) and secretion of the drug into the tubules.

In the presence of risk factors such as CKD, treatment becomes challenging [5]. In patients with CKD, the glomerular filtration rate may vary [3]. Quinolones and cotrimoxazole are found to reach the renal tissue and urine in adequate concentrations; hence are widely used as treatment options for UTI in CKD. The drug dosage however is modified based on the estimated GFR of the patient [3].

The pattern of microbiological profile and antibiotic sensitivity is constantly changing worldwide [5] . Escherichia coli (E. Coli) has been found to be the most common pathogen worldwide for both outpatients and inpatients [3]. Major problem in recent times is the abuse of antibiotics, which leads to emergence of multi-drug resistance [7]. It is important for us to study the microbiological profile and antibiotic sensitivity pattern in our tertiary care centre to formulate treatment guidelines for empirical antibiotics while awaiting urine culture reports [7]. 

Hence, our objective was to study the microbiological and antibiotic sensitivity pattern of UTI in CKD patients in a tertiary health care centre.

## Materials and methods

This was a retrospective descriptional study conducted at Institute of Nephro-Urology, Bengaluru, which is a tertiary government-run referral hospital catering to patients with renal problems. We studied 129 CKD patients with signs and symptoms of UTI over a period of six months (from December 2019 to May 2020).

Inclusion criteria

(1) Patients aged above 18 years (both males and females), (2) patients with CKD stage 1 to stage 5 according to Kidney Disease Outcomes Quality Initiative (KDOQI) clinical practice guidelines, including patients on dialysis, (3) patients having symptoms of UTI-like burning micturition, increased frequency or urgency of urination, abdomen or loin pain and fever, and (4) urine culture positive for at least one bacterial strain.

Exclusion criteria

(1) Renal transplant patients, (2) patients on immunosuppressive therapy for any other primary systemic disease or primary glomerular disease, (3) patients who had taken antibiotics 48 hours prior to urine culture specimen collection, and (4) sterile urine cultures.

Sample collection

10 ml of clean-catch midstream urine sample was collected by the patients into a universal, wide-mouth, sterile, leakproof container provided by the lab after thorough cleaning of the external genitalia with soap and water [8]. The samples were cultured within two hours on to Cystine-Lactose-Electrolyte-Deficient (CLED) medium by Mayo’s semiquantitative method and incubated overnight at 37 degrees Celcius. The colony count were enumerated as per the standard guidelines as significant (>105 cfu/ml), probably significant (1000-100000 cfu/ml) and insignificant (<1000 cfu/ml) [9]. The isolates were identified using standard biochemical tests [10].

Antibiotic susceptibility test was performed and interpreted using the Kirby-Bauer disc diffusion method on Mueller Hinton agar medium according to Clinical and Laboratory Standards Institute guidelines [11].

Microscopic analysis of fresh urine sample was done after centrifuging it at 2000 rpm for 15 minutes. Blood samples from each patient were analyzed for complete blood count and renal function tests based on standard guidelines [12].

Statistical methods

SPSS version 20.0 software (IBM Corp, Armonk, NY) was used to carry out statistical methods. Microsoft Excel and Word (Microsoft Corporation, Redmond, WA) were used to prepare graphs and tables. Descriptive statistics such as mean, standard deviation, proportion (%) were calculated using SPSS software.

## Results

Among 129 CKD patients, UTI was most common between 61 to 70 years of age (25.58%), followed by 51-60 years (19.3%), 51-50 years (13.17%), 31-40 years (12.40%), 71-80 years (10.07%), 20-30 years (8.52%), more than 80 years (5.42%). The mean age was 55.91+17 years. Out of the total number of patients studied, 76.2% were males and 23.8% were females.

The most common presenting symptoms were urinary complaints (63%) such as burning micturition (dysuria), increased frequency of micturition followed by fever (23%), pain abdomen(11%), and other nonspecific complaints (3%).

11 patients (9%) had a growth of two microorganisms and the rest 118 (91%) had a growth of a single organism. The total number of microorganisms grown was 131. 123 (94%) were gram positive, four (3%) were gram negative, four (3%) were *Candida *species [Table 1]

**Table 1:** Organism distribution studied

Organism	No. of patients	%
GRAM-NEGATIVE BACTERIA		
Escherichia coli	81	61.8
Klebsiella species	18	13.74
Pseudomonas species	10	7.6
Citrobacter diversus	7	5.3
Nonfermenting gram-negative bacilli	3	2.2
Serratia marcescens	2	1.52
Proteus mirabilis	1	0.76
Enterobacter	1	0.76
GRAM-POSITIVE BACTERIA		
Enterococcus	3	2.29
Staphylococcus aureus	1	0.76
FUNGAL SPECIES		
Candida albicans	2	1.52
Non-Candida albicans	2	1.52
TOTAL	131	100

*E. coli* (61.8%) was the most common culture grown organism, followed by *Klebsiella* (13.74%) and *Pseudomonas* (7.6%). The most common gram-positive organism isolated was *Enterococcus* (2.29%), followed by *Staphylococcus aureus *(0.76%) [Table 1].

*E. coli *was the most common isolate among all age groups. *Klebsiella *species was the second-most common isolate found in younger adults, whereas *Pseudomonas *species was the second-most common isolate among the elderly [Table 2].

**Table 2:** Organism distribution of patients studied in relation to the age of patients studied

Organism	Age in years						Total	
	20-30yrs	31-40yrs	41-50yrs	51-60yrs	61-70yr	More than 70yrs		Percentage %
E.Coli	5(45.45%)	7 (43.75%)	12( 60%)	20(68.96%)	23(65.71%)	14 (66.66%)	81	61.8
Klebsiella	3(27.27%)	3(18.75%)	1(5%)	6(20.68%)	2(5.71%)	3(14.28%)	18	13.74
Pseudomonas	1(9.09%)	1(6.25%)	4(20%)	0	4(11.42%)	0	10	7.6
Citrobacter diversus	2(18.18%)	2(12.5%)	1(5%)	1(3.44%)	0	1(4.76%)	7	5.3
Nonfermenting gram-negative bacilli	0	0	0	0	2(5.71%)	1(4.76%)	3	2.2
Serratia marcescens	0	1(6.25%)	1(5%)	0	0	0	2	1.52
Proteus mirabilis	0	0	0	1(3.44%)	0	0	1	0.76
Enterobacter	0	0	0	0	1(2.8%)	0	1	0.76
Enterococcus	0	1(6.25%)	0	0	1(2.8%)	1(4.76)	3	2.29
Coagulase-negative Staphylococcus aureus	0	1(6.25%)	0	0	0	0	1	0.76
Candida species	0	0	0	1(3.44%)	2(5.71%)	1(4.76%)	4	3.04
TOTAL	11	16	20	29	35	21	131	100

*E. coli *was grown in 65.69% in males and 52.63% in females respectively [Table 3]. Hence, it was the most common microorganism isolated irrespective of gender.

**Table 3:** Organism distribution of patients studied in relation to the gender of patients studied

Organism	Gender		Total
	Female	Male	
E. coli	20 (52.63%)	61(65.69%)	81
Klebsiella	6(15.7%)	12(12.90%)	18
Pseudomonas	3(7.8%)	7(7.5%)	10
Citrobacter diversus	1(2.6%)	6(6.4%)	7
Nonfermenting gram-negative bacilli	2(5.2%)	1(1.07%)	3
Serratia marcescens	1(2.6%)	1(1.07%)	2
Proteus mirabilis	1(2.6%)	0	1
Enterobacter	0	1(1.07%)	1
Enterococcus	1(2.6%)	2(2.15%)	3
Coagulase-negative Staphylococcus aureus	0	1(1.07%)	1
Candida species	3(7.89%)	1(1.07%)	4
TOTAL	38	93	131

The overall antibiotic sensitivity pattern for gram-negative organisms showed maximum sensitivity for fosfomycin (100%), colistin (100%), tigecycline (100%), polymyxin B (100%), followed by meropenem (95.2%), imepenem (93.6%), and amikacin (87.2%) [Table 4].

**Table 4:** Overall antibiotics resistance/susceptibility

Antibiotics	Intermediate	Resistant	Sensitive
Amikacin	1(0.8%)	15(12%)	109(87.2%)
Gentamicin	2(1.6%)	37(29.6%)	86(68.8%)
Amoxicillin-clavulanic acid	10(8%)	85(68%)	30(24%)
Aztreonam	0(0%)	81(64.8%)	44(35.2%)
Cefotaxime	0(0%)	86(68.8%)	39(31.2%)
Ceftazidime	0(0%)	83(66.4%)	42(33.6%)
Ciprofloxacin	8(6.4%)	90(72%)	27(21.6%)
Norfloxacin	0(0%)	86(68.8%)	39(31.2%)
Levofloxacin	2(1.6%)	75(60%)	48(38.4%)
Nitrofurantoin	2(1.6%)	38(30.4%)	85(68%)
Ertapenem	1(0.8%)	18(14.4%)	106(84.8%)
Imipenem	1(0.8%)	7(5.6%)	117(93.6%)
Meropenem	0(0%)	6(4.8%)	119(95.2%)
Cefoperazone-sulbactam	11(8.8%)	17(13.6%)	97(77.6%)
Piperacillin-tazobactam	11(8.8%)	17(13.6%)	97(77.6%)
Polymyxin-B	0(0%)	0(0%)	125(100%)
Colistin	0(0%)	0(0%)	125(100%)
Tigecycline	0(0%)	0(0%)	125(100%)
Fosfomycin	0(0%)	0(0%)	125(100%)
Doxycycline	0(0%)	41(32.8%)	84(67.2%)
Cotrimoxazole	0(0%)	63(50.4%)	62(49.6%)

Maximum resistance of all the organisms including *E. coli* was for ciprofloxacin (72%), followed by norfloxacin (68.8%), levofloxacin (60%). *E. coli* and *Klebsiella *were resistant to amoxicillin-clavulanic acid by 71.6% and 72.2% respectively. *Klebsiella *was also resistant to nitrofurantoin, cotrimoxazole, and third-generation cephalosporins. Resistance to third-generation cephalosporins was due to extended-spectrum beta-lactamase [ESBL] production. 77.1% of *E. coli *and 61.1% of *Klebsiella *were ESBL-producing microorganisms [Table 5].

**Table 5:** Antibiotics: resistance pattern for gram-negative organisms

Antibiotics									
	Citrobacter diversus (n=7)	E. coli (n=81)	Enterobacter (n=1)	Klebsiella (n=18)	Nonfermenting gram-negative bacilli (n=3)	Proteus mirabilus (n=1)	Pseudomonas (n=10)	Serratia marscesans (n=2)	
Amikacin	1(14.3%)	6(7.4%)	1(100%)	4(22.2%)	0(0%)	0(0%)	2(20%)	0(0%)	
Gentamicin	5(71.4%)	24(29.6%)	1(100%)	4(22.2%)	0(0%)	0(0%)	3(30%)	0(0%)	
Amoxicillin-clavulanic acid	6(85.7%)	58(71.6%)	1(100%)	13(72.2%)	2(66.7%)	1(100%)	NA	1(50%)	
Aztreonam	4(57.1%)	63(77.8%)	1(100%)	11(61.1%)	0(0%)	1(100%)	1(10%)	0(0%)	
Cefotaxime	5(71.4%)	63(77.8%)	1(100%)	11(61.1%)	1(33.3%)	1(100%)	NA	0(0%)	
Ceftazidime	4(57.1%)	63(77.8%)	1(100%)	11(61.1%)	0(0%)	1(100%)	1(10%)	0(0%)	
Ciprofloxacin	3(42.9%)	69(85.2%)	0(0%)	11(61.1%)	0(0%)	1(100%)	4(40%)	1(50%)	
Norfloxacin	2(28.6%)	70(86.4%)	0(0%)	9(50%)	0(0%)	1(100%)	2(20%)	0(0%)	
Levofloxacin	1(14.3%)	63(77.8%)	0(0%)	9(50%)	0(0%)	1(100%)	1(10%)	0(0%)	
Nitrofurantoin	5(71.4%)	16(19.8%)	0(0%)	13(72.2%)	1(33.3%)	0(0%)	NA	2(100%)	
Ertapenem	1(14.3%)	11(13.6%)	0(0%)	4(22.2%)	0(0%)	0(0%)	NA	0(0%)	
Imipenem	0(0%)	4(4.9%)	0(0%)	3(16.7%)	0(0%)	0(0%)	0(0%)	0(0%)	
Meropenem	0(0%)	2(2.5%)	0(0%)	4(22.2%)	0(0%)	0(0%)	0(0%)	0(0%)	
Cefoperazone -sulbactam	0(0%)	12(14.8%)	0(0%)	4(22.2%)	0(0%)	0(0%)	1(10%)	0(0%)	
Piperacillin-tazobactam	0(0%)	13(16%)	0(0%)	4(22.2%)	0(0%)	0(0%)	0(0%)	0(0%)	
Polymyxin-B	0(0%)	1(1.2%)	0(0%)	0(0%)	0(0%)	0(0%)	0(0%)	0(0%)	
Colistin	0(0%)	1(1.2%)	0(0%)	0(0%)	0(0%)	0(0%)	1(10%)	0(0%)	
Tigecycline	0(0%)	1(1.2%)	0(0%)	0(0%)	0(0%)	0(0%)	NA	0(0%)	
Fosfomycin	0(0%)	0(0%)	0(0%)	0(0%)	1(33.3%)	0(0%)	NA	0(0%)	
Doxycycline	3(42.9%)	30(37%)	0(0%)	4(22.2%)	2(66.7%)	0(0%)	NA	0(0%)	
Cotrimoxazole	5(71.4%)	43(53.1%)	1(100%)	10(55.6%)	1(33.3%)	1(100%)	NA	0(0%)	

*Pseudomonas* was highly sensitive to aminoglycosides, ceftazidime, cefoperazone-sulbactam, levofloxacin, and norfloxacin. It showed 100% susceptibility to piperacillin-tazobactam, carbapenems, polymyxin-B, and colistin. All the gram-negative organisms, including *E. coli*, showed a high percentage of sensitivity to cefoperazone-sulbactam, piperacillin-tazobactam, carbapenems, fosfomycin, tigecycline, polymyxin-B, and colistin [Table 6].

**Table 6:** Antibiotics: sensitivity pattern for gram-negative organisms

Antibiotics									
	Citrobacter diversus (n=7)	E. coli (n=81)	Enterobacter (n=1)	Klebsiella (n=18)	Nonfermenting gram-negative bacilli (n=3)	Proteus mirabilus (n=1)	Pseudomonas (n=10)	Serratia marscesans (n=2)	
Amikacin	6(85.7%)	75(92.6%)	0(0%)	14(77.8%)	3(100%)	1(100%)	7(70%)	2(100%)	
Gentamicin	2(28.6%)	55(67.9%)	0(0%)	14(77.8%)	3(100%)	1(100%)	7(70%)	2(100%)	
Amoxicillin-clavulanic acid	0(0%)	14(17.3%)	0(0%)	5(27.8%)	1(33.3%)	0(0%)	NA	1(50%)	
Aztreonam	3(42.9%)	18(22.2%)	0(0%)	7(38.9%)	3(100%)	0(0%)	9(90%)	2(100%)	
Cefotaxime	2(28.6%)	18(22.2%)	0(0%)	7(38.9%)	2(66.7%)	0(0%)	NA	2(100%)	
Ceftazidime	3(42.9%)	18(22.2%)	0(0%)	7(38.9%)	3(100%)	0(0%)	9(90%)	2(100%)	
Ciprofloxacin	2(28.6%)	8(9.9%)	0(0%)	6(33.3%)	3(100%)	0(0%)	6(60%)	1(50%)	
Norfloxacin	5(71.4%)	11(13.6%)	1(100%)	9(50%)	3(100%)	0(0%)	8(80%)	2(100%)	
Levofloxacin	6(85.7%)	16(19.8%)	1(100%)	9(50%)	3(100%)	0(0%)	9(90%)	2(100%)	
Nitrofurantoin	2(28.6%)	65(80.2%)	1(100%)	4(22.2%)	2(66.7%)	1(100%)	NA	0(0%)	
Ertapenem	6(85.7%)	70(86.4%)	1(100%)	14(77.8%)	2(66.7%)	1(100%)	NA	2(100%)	
Imipenem	7(100%)	77(95.1%)	1(100%)	14(77.8%)	3(100%)	1(100%)	10(100%)	2(100%)	
Meropenem	7(100%)	79(97.5%)	1(100%)	14(77.8%)	3(100%)	1(100%)	10(100%)	2(100%)	
Cefoperazone -sulbactam	7(100%)	58(71.6%)	1(100%)	14(77.8%)	3(100%)	1(100%)	9(90%)	2(100%)	
Piperacillin-tazobactam	7(100%)	58(71.6%)	1(100%)	13(72.2%)	3(100%)	1(100%)	10(100%)	2(100%)	
Polymyxin-B	7(100%)	80(98.8%)	1(100%)	18(100%)	3(100%)	1(100%)	10(100%)	2(100%)	
Colistin	7(100%)	80(98.8%)	1(100%)	18(100%)	3(100%)	1(100%)	9(90%)	2(100%)	
Tigecycline	7(100%)	80(98.8%)	1(100%)	18(100%)	3(100%)	1(100%)	NA	2(100%)	
Fosfomycin	7(100%)	81(100%)	1(100%)	18(100%)	2(66.7%)	1(100%)	NA	2(100%)	
Doxycycline	4(57.1%)	51(63%)	1(100%)	14(77.8%)	1(33.3%)	1(100%)	NA	2(100%)	
Cotrimoxazole	2(28.6%)	38(46.9%)	0(0%)	8(44.4%)	2(66.7%)	0(0%)	NA	2(100%)	

Antibiotic sensitivity pattern for gram-positive organisms showed a 100% sensitivity to vancomycin, cotrimoxazole, linezolid, teicoplanin [Table 7].

**Table 7:** Antibiotics: sensitivity pattern for gram-positive organisms

Gentamicin	Ciprofloxacin	Levofloxacin	Penicillin	Nitrofuratoin	Doxycycline	Vancomycin	Teicoplanin	Linezolid	Cotrimoxazole	Ciprofloxacin	Ampicillin
100%	50%	50%	0%	75%	25%	100%	100%	100%	100%	100%	66.60%

56.6% of the patients with culture-positive urinary tract infections had 0 to 5 pus cells. Significant pyuria (white blood cells (WBC) >5 per high power field) was seen in only 39.6% of the cases. 60.4% of cases did not show any significant pyuria. 

59.7% of the patients had a total leucocyte count between 4000-11000/mm^3^. 3.9% of the patients had a total leucocyte count less than 4000/mm^3^. 33.3% had a total count of more than 11000/mm^3^.

## Discussion

We have studied the clinical presentation, microorganisms and their antibiotic sensitivity pattern in 129 culture-proven urinary tract infections in the CKD population presenting to a tertiary care centre.

As per a study by Ronald et al [13], females are more commonly infected compared to males. Patel et al [14] studied patients with urinary tract infection in Gujarat and found that the culture positivity was more in females compared to males. Females tend to acquire infection more easily than men due to short urethra and closer position of the urethra with the rectum. However, in our study, males were commonly affected. This can be attributed to the increased incidence of CKD in males compared to females.

Elderly patients (between 61 to 70 years followed by 51 to 60 years) were most commonly affected. This result is compatible with a study by Eshwarappa et al [15]. The elderly are more prone to urinary tract infections. Urinary retention and high post-void residue are risk factors for infection in this age group. Prostate hypertrophy and autonomic neuropathy are the most common causes of urinary stasis [16].

The most common symptom was dysuria and other urinary symptoms such as urinary incontinence, increased frequency, macrohaematuria, followed by fever and suprapubic pain in our study, which is similar to a systematic review by Schmiemann et al [17]. Few of the patients were asymptomatic, yet they had culture-positive urinary tract infection with a significant bacterial count. Symptoms play a minor role in the diagnosis of urinary tract infection. A combination of two or more symptoms such as dysuria and fever may be more predictive of urinary tract infection than just a single symptom [18].

Gram-negative organisms, especially *E. coli*, were the most common organism isolated in our study, followed by *Klebsiella *species. Very few gram-positive organisms were isolated, most common being *Enterococcus *followed by *Staphylococcus aureus *[Table 1]. These results are similar to the results from other studies of community-acquired UTI [19,20].

Antibiotic resistance is a major increasing problem worldwide in treating infections. The organisms from Latin America are the most resistant followed by the Asia Pacific and European countries. The antibiotic resistance is least in Canada [20]. In our study, antibiotic resistance was maximum for quinolones [Table 4]. The maximum resistance of all the organisms including *E. coli *was for ciprofloxacin (72%), followed by norfloxacin (68.8%), and levofloxacin (60%) [Table 5]. These results are similar to the study by Eshwarappa et al [15]. *Klebsiella *was grown in only 13.74% of the urine cultures compared to *E. coli, *which was grown in 61.8%. *Klebsiella *species is not a common cause of community-acquired UTI [21]. These results are similar to a study by Akram et al [21]. *E. coli *and *Klebsiella *were resistant to amoxicillin-clavulanic acid by 71.6% and 72.2% respectively. *Klebsiella *was also resistant to nitrofurantoin, cotrimoxazole, and third-generation cephalosporins. Resistance to third-generation cephalosporins was due to extended spectrum beta-lactamase [ESBL] production. 77.1% of *E. coli *and 61.1% of *Klebsiella *were ESBL-producing microorganisms [Table 5]. High resistance rates among all these organisms to most of the antibiotics except carbapenems shows the inadvertent use of antibiotic in the past decades. Multi-drug-resistant strains of *E. coli *are common [22]. These results are similar to community studies over the past few years [23].

*Pseudomonas *was highly sensitive to aminoglycosides, ceftazidime, cefoperazone-sulbactam, levofloxacin, and norfloxacin [Table 5]. It showed 100% susceptibility to piperacillin-tazobactam, carbapenems, polymyxin-B, and colistin. All the gram-negative organisms, including *E. coli, *showed a high percentage of sensitivity to cefoperazone-sulbactam, piperacillin-tazobactam, carbapenems, fosfomycin, tigecycline, polymyxin-B, and colistin [Table 6]. These results are similar to the study by Akram et al [21]. Carbapenem resistance was seen in only 5 to 6% in our study, indicating good antibiotic prescription practice at our institute.

Gram-positive isolates were very rare compared to gram-negative isolates. The most common gram-positive isolate was *Enterococcus* species [75%], followed by *Staphylococcus aureus *[25%]. They showed resistance to quinolones, penicillin, and doxycycline. They were highly sensitive to vancomycin, linezolid, and teicoplanin [Table 7]. The high degree of resistance to penicillin may be due to beta-lactamase production [23].

## Conclusions

There are very few studies on urinary tract infection in the CKD population. The CKD population is immunocompromised and is more prone to infection due to chronic inflammation. It is alarming to note that these microorganisms were resistant to at least two or more groups of antibiotics. This emphasizes the need of the hour, which is to formulate a uniform empirical antibiotic policy for better management and clinical outcome of the patients with UTI in the CKD population.
